# The predictive value of NLR, SII, and complement 3 in treatment response for systemic lupus erythematosus with immune thrombocytopenia

**DOI:** 10.3389/fimmu.2025.1606510

**Published:** 2025-07-29

**Authors:** Ziqiang Zheng, Jiali Liu, Taoyuan He, Chang Liu

**Affiliations:** ^1^ Department of Rheumatology and Immunology, Central Hospital of Dalian University of Technology, Dalian, China; ^2^ Graduate School, Dalian Medical University, Dalian, China; ^3^ Department of Endocrinology, Central Hospital of Dalian University of Technology, Dalian, China

**Keywords:** systemic lupus erythematosus, immune thrombocytopenia, systemic immune-inflammation index, neutrophil-to-lymphocyte ratio, treatment

## Abstract

**Objective:**

To evaluate the predictive value of systemic immune-inflammation index (SII), neutrophil-to-lymphocyte ratio (NLR), and complement 3 in the therapeutic outcomes of systemic lupus erythematosus-associated immune thrombocytopenia (SLE-ITP).

**Methods:**

Clinical data from 72 patients diagnosed with SLE-ITP and admitted to the Affiliated Central Hospital of Dalian University of Technology between January 2013 and September 2024 were collected and analyzed. Associations between therapeutic outcomes and clinical characteristics, as well as laboratory findings including SII and NLR, were evaluated systematically.

**Results:**

The patients achieved complete remission (CR) 32 (44.4%), partial remission (PR) 19 (26.4%), and no remission (NR) 21 (29.2%). SII exhibited statistically significant differences among the three groups (*P*=0.027). The median SII values were 145.7 (43.7-238.7) for the CR group, 57.2 (20.5-90.2) for the PR group, and 117.8 (80.7-238.6) for the NR group. Additionally, complement 3 levels were significantly lower in the CR group compared to the PR and NR groups (*P*=0.010). Logistic regression analysis revealed that the NLR was positively correlated with therapeutic efficacy (OR=1.982, 95% CI: 1.18-3.33, *P*=0.010). SII and complement 3 were significantly negatively correlated with therapeutic outcomes (SII: OR=0.991, 95% CI: 0.984-0.998, *P*=0.011; C3: OR=0.045, 95% CI: 0.002-0.919, *P*=0.044). ROC curve analysis demonstrated that the combined predictive model of NLR, SII, and complement 3 achieved an area under the curve (AUC) of 0.743 (95% CI: 0.620-0.866, *P*=0.001), specificity of 76.2%, and sensitivity of 66.7%, indicating excellent predictive efficacy.

**Conclusion:**

The combined predictive model significantly enhances the predictive efficacy for SLE-ITP treatment outcomes compared to individual indicators.

## 1 Introduction

Systemic Lupus Erythematosus (SLE) is an inflammatory autoimmune disease characterized by multi-system involvement. Its pathological features include the production of autoantibodies and the deposition of immune complexes, resulting in damage to multiple organ systems, particularly hematological abnormalities (1, 2). Epidemiological data indicate that thrombocytopenia is the most common hematological manifestation of SLE, with an incidence rate of up to 56.1% (3). Systemic Lupus Erythematosus-Associated Immune Thrombocytopenia (SLE-ITP) is typically defined as immune-mediated thrombocytopenia driven by autoimmune mechanisms of SLE, with peripheral blood platelet count below 100×109/L, after the exclusion of other potential causes, such as drug-induced effects or thrombotic microangiopathy syndrome (4, 5). Immune thrombocytopenia (ITP) exhibits heterogeneous clinical manifestations, with marked variations in disease progression and symptom severity among patients. ITP may present as either acute or chronic, and some cases progress to severe thrombocytopenia. Studies indicate that approximately 3%-20% of ITP patients meet the diagnostic criteria for severe thrombocytopenia—a condition linked to prolonged SLE course (6). When SLE involves severe hematological manifestations, the risk of cardiovascular complications also rises significantly (7). Further analysis reveals that, compared to primary ITP, SLE-ITP demonstrates a higher incidence of major bleeding events (19% vs. 10%, respectively) (6). Therefore, SLE-ITP usually indicates heightened disease activity, which not only significantly increases the risk of end-organ damage but is also strongly associated with poor prognosis and elevated mortality rates.

Similar to the therapeutic approach for primary immune thrombocytopenia, the treatment of SLE-ITP primarily relies on immunosuppressive agents (8). Studies have shown that approximately 75% of SLE-ITP patients are initially treated with glucocorticoids in combination with antimalarial drugs (6). However, about 30% of patients exhibit resistance to this regimen (9). To increase platelet counts and prevent life-threatening bleeding events in SLE-ITP, high-dose glucocorticoids combined with immunosuppressants are commonly used as an intensive therapeutic approach. Nevertheless, long-term use of such treatments may result in immunosuppression, increased risk of opportunistic infections, and drug-related adverse effects, such as renal or hepatic impairment, which adversely impact overall prognosis, quality of life, and significantly increase the financial burden on patients (10, 11).

Given the complexity of the pathogenesis and the uncertainty of prognosis in SLE-ITP, identifying and validating clinical parameters or biomarkers for predicting therapeutic outcomes is of significant clinical importance. This approach could effectively reduce unnecessary therapeutic interventions, optimize individualized treatment regimens, lower the risk of infections and drug-related adverse effects, and ultimately achieve early remission and improve long-term patient outcomes. Therefore, our retrospective study evaluated whether systemic immune-inflammation index (SII) and neutrophil-to-lymphocyte ratio (NLR), derived from peripheral blood inflammation parameters, could serve as predictive factors for immunotherapy response.

## 2 Materials and methods

We retrospectively collected and analyzed data from inpatients diagnosed with SLE at the Affiliated Central Hospital of Dalian University of Technology from January 2013 to September 2024. Inclusion criteria were based on the 1997 American College of Rheumatology (ACR) classification criteria (12) or the 2019 European League Against Rheumatism (EULAR)/ACR classification criteria (13) for SLE. Patients with other hematological disorders, such as myelodysplastic syndromes (MDS), aplastic anemia (AA), thrombotic microangiopathy (TMA), thrombotic thrombocytopenic purpura (TTP), and hypersplenism, leading to thrombocytopenia were excluded. A total of 72 cases of SLE-ITP were included in the study.

Patient data, including demographic characteristics, clinical manifestations, laboratory test results, and imaging findings, were systematically collected and analyzed. Parameters included lymphocyte subpopulations, SII(platelet count ×neutrophil/lymphocyte count at diagnosis) (14), platelet-to-lymphocyte ratio(PLR), NLR, and treatment regimens. All predictive indicators(including the data presented in **Table 1**) - SII, NLR, PLR, complement levels blood counts, and lymphocyte subsets - were collected as baseline data at the time of hospital admission for SLE-ITP diagnosis and before initiating any immunomodulatory therapy (i.e., pre-treatment intervention). Ethical approval for this retrospective study was obtained from the Institutional Review Board of the Affiliated Central Hospital of Dalian University of Technology. As the study was based on the review of medical records for clinical purposes, written informed consent was waived. Patient information was anonymized and de-identified before analysis.

**Table 1:** Analysis of clinical characteristics in 72 patients with SLE-ITP.

Variables	CR+PR,n=51	CR,n=32	PR,n=19	NR,n=21	*P*1	*P*2
Age, years	44.6 ± 16.9	43.75 ± 16.56	46 ± 17.83	53.2 ± 15.1	0.124	0.046*
Female (%)	43(84.3)	28(87.5)	15(78.9)	15(71.4)	0.331	0.353
Rash (%)	15(29.4)	12(37.5)	3(15.8)	4(19)	0.156	0.364
Arthritis (%)	18(35.3)	12(37.5)	6(31.6)	5(23.8)	0.578	0.342
Alopecia (%)	9(17.6)	9(28.1)	0	3(14.3)	0.023*	1.000
Nephritis (%)	22(43.1)	16(50)	6(31.6)	3(14.3)	0.024*	0.019*
WBC, ×10^9^/L	4.5(2.7,6.7)	3.3(2.6,5.9)	5.9(3.4,9.6)	3.4(2.4,4.9)	0.112	0.252
Neut, ×10^9^/L	2.8(1.6,4.8)	2.3(1.6,4.1)	3.5(2.1,5.7)	2(1.6,3.9)	0.093	0.165
Lym, ×10^9^/L	1(0.8,1.4)	0.91(0.6,1.5)	1(0.8,1.4)	0.95(0.6,1.5)	0.799	0.857
HB, g/l	104.8 ± 24.2	101 ± 22.1	111.3 ± 26.6	108.8 ± 26.9	0.303	0.540
ALT, U/L	21(13,38)	29(13.2,38.7)	15(10,27)	18(14,30.5)	0.170	0.590
AST, U/L	24(19,38)	27(21.2,58)	21(16,29)	20(17,33.5)	0.121	0.857
ALP, U/L	73(57,87)	78.5(58,88.7)	68(54,81.2)	74(58,89.5)	0.544	0.896
GGT, U/L	25(15,34)	25.5(15,37)	25(15,29)	25(12.5,40)	0.785	0.624
Na+,mmol/L	137.8 ± 3.6	137.1 ± 3.8	138.8 ± 3.1	138.1 ± 3.3	0.279	0.724
FIB,g/L	3.4(2.7,3.6)	3.3(2.6,3.4)	3(2.8,3.9)	3.1(2.8,3.5)	0.370	0.935
C3,g/l	0.6 ± 0.1	0.5 ± 0.2	0.7 ± 0.2	0.6 ± 0.2	0.010*	0.356
Platelet,×10^9^/L	39(8,69)	59.5(19.8,82.3)	18(6,39)	63(39.5,77.5)	0.001*	0.031*
NLR	2.8(1.9,5.2)	2.2(1.8,5.8)	3.4(2.1,5.1)	2.2(1.5,3.3)	0.174	0.173
PLR	32.1 (9.8,76.3)	48.9 (16.9,94.8)	13.5 (4.2,13.5)	60 (32.2,98.7)	0.002*	0.036*
SII	81.6 (31,187)	145.7 (43.7,238.7)	57.2 (20.5,90.2)	117.8 (80.7,238.6)	0.027*	0.187
Anti-dsDNA, IU/ml	508(240,800)	585(313,800)	340(240,600)	306(181,462)	0.064	0.087
Anti-SM(%)	8(15.7)	6(18.8)	2(10.5)	0	0.09	0.13
SLEDAI	11(6,18)	12.5(7,18)	9(5,12)	6(4,15.5)	0.152	0.228

*P < 0.05. *P*1 represents the analysis of differences among the CR, PR, and NR groups, while *P*2 represents the analysis of differences between the CR+PR and NR groups. FIB: fibrinogen, C3: complement 3, SLEDAI: systemic Lupus Erythematosus Disease Activity Index, PLR (platelet-to-lymphocyte ratio), NLR (neutrophil-to-lymphocyte ratio), SII (systemic immune-inflammation index, platelet count × neutrophil count/lymphocyte count).

Clinical response definitions included: CR: platelet count persistently ≥100×10^9/L on at least two consecutive occasions. PR: platelet count increased from 20–50×10^9/L to 50–100×10^9/L or from <20×10^9/L to above 20×10^9/L on two consecutive occasions (15, 16). NR: failure to meet CR or PR criteria. Patients achieving CR or PR were classified as treatment responders, and the treatment-effective group was defined as CR + PR. Concerning the definition of CR or PR, at least two separate platelet counts (with a minimum interval of 7 days) were required for confirmation (17). The minimum platelet count before initiating glucocorticoid and immunosuppressive therapy was recorded as the baseline platelet count. All laboratory tests were completed within 24 hours before treatment initiation. Glucocorticoids were administered for active SLE-ITP or visceral involvement (e.g., lupus nephritis), and platelet transfusions were considered for patients with platelet counts <30×10^9^/L or active bleeding (18).

Statistical Analysis: Normally distributed continuous variables were expressed as mean ± standard deviation and compared using t-tests or analysis of variance (ANOVA) for two or three groups, respectively. Non-normally distributed continuous variables were presented as median (interquartile range [IQR]) and analyzed using non-parametric tests, such as the Mann-Whitney U test or Kruskal-Wallis test. Categorical variables were expressed as counts (%) and compared using Chi-squared tests or Fisher’s exact tests. Univariate and multivariate logistic regression analyses were performed to evaluate the associations between lymphocyte subpopulations, inflammation indices (SII, PLR, NLR), and treatment response, with NR as the reference group. Receiver operating characteristic (ROC) curve analysis determined the optimal cutoff values for sensitivity and specificity. P-value <0.05 was considered statistically significant. Statistical analyses were performed using SPSS 25.0, and ROC curves were generated using GraphPad Prism 9.5.

## 3 Results

### 3.1 Baseline characteristics

A total of 72 patients were included in this study, of whom 58 (80.6%) were female. The mean age was 47.1 ± 16.8 years. Analysis of disease duration revealed a median SLE duration of 12 (interquartile range, 2.3–72.0) months and a median ITP duration of 2 (interquartile range, 0.3–49.5) months. Peripheral blood inflammation-derived indices demonstrated the following results: median NLR of 2.6 (1.9–3.9), median PLR of 38.8 (14.0–87.9), and median SII of 93.6 (45.7–195.9). Detailed baseline characteristics are presented in **Table 2**.

**Table 2:** Baseline characteristics of 72 patients with SLE-ITP.

Characteristics	Values
Demographic Data	
Age, years	47.1 ± 16.8
Sex, female (%)	58(80.6%)
SLE Duration, months	12(2.3,72.0)
ITP Duration, months	2(0.3,49.5)
Laboratory Tests	
WBC,×10^9^/L	4(2.7,6.5)
Neut,×10^9^/L	2.4(1.6,4.6)
Lym,×10^9^/L	0.9(0.7,1.4)
Initial Platelet Count,×10^9^/L	47(18.3,74.3)
Hb,g/l	106 ± 24.9
NLR	2.6(1.9,3.9)
PLR	38.8 (14.0,87.9)
SII	93.6 (45.7,195.9)
ALT,U/L	20.5(13,33.8)
AST,U/L	24(19,34.8)
ALP,U/L	73(58,86.3)
GGT,U/L	25(15,37)
Na+,mmol/l	137.9 ± 3.6
TC,mmol/l	4.6(3.5,4.6)
TG,mmol/l	1.3(1.1,1.6)
HDL-C,mmol/l	1(0.9,1.1)
LDL-C,mmol/l	2.2(2,2.5)

WBC (white blood cell count), Neut (neutrophil count), Lym (lymphocyte count), HB (hemoglobin), PLR (platelet-to-lymphocyte ratio), NLR (neutrophil-to-lymphocyte ratio), SII (systemic immune-inflammation index, platelet count × neutrophil count/lymphocyte count), ALT (alanine aminotransferase), AST (aspartate aminotransferase), ALP (alkaline phosphatase), GGT (gamma-glutamyl transferase).

### 3.2 Analysis of patient clinical characteristics

Based on therapeutic outcomes, the 72 patients were categorized into three groups: CR in 32 cases (44.4%), PR in 19 cases (26.4%), and NR in 21 cases (29.2%). For further analysis, the CR and PR groups were combined into a treatment-responsive group, comprising 51 cases (70.8%). Age analysis demonstrated significant differences, with the NR group having a mean age of 53.2 ± 15.1 years, compared to 44.6 ± 16.9 years in the CR+PR group (*P*=0.046). Compared with the NR group, the CR+PR group exhibited a significantly lower median baseline platelet count, with the difference reaching statistical significance (*P*=0.031).

Analysis of inflammation-derived indices revealed the following: The PLR was 48.9(16.9, 94.8) in the CR group and 13.5(4.2, 13.5) in the PR group, both significantly lower than the PLR of 60.0(32.2, 98.7) in the NR group (*P*=0.002). Further analysis demonstrated that the PLR in the CR+PR group (32.1) was significantly lower compared to the NR group (60.0) (*P*=0.036). The SII was 145.7(43.7, 238.7) in the CR group, 57.2(20.5, 90.2) in the PR group, and 117.8(80.7, 238.6) in the NR group, with statistically significant differences among the three groups (*P*=0.027). Although the median SII in the CR+PR group was lower than that in the NR group, the difference was not statistically significant (*P*=0.187). Detailed results are summarized in **Table 1**.

In addition to the aforementioned clinical characteristics, significant differences were observed in treatment regimens among the groups. Glucocorticoid usage was reported in 27 cases (84.4%) of the CR group and 16 cases (84.2%) of the PR group, both significantly higher than 9 cases (42.9%) in the NR group (*P*=0.002). Similarly, the combined CR+PR group exhibited significantly higher glucocorticoid usage compared to the NR group (*P*<0.001). The use of hydroxychloroquine was also significantly higher in the CR+PR group compared to the NR group (*P*=0.034). Detailed treatment regimens are presented in **Table 3**.

**Table 3:** Analysis of treatment regimens in 72 patients with SLE-ITP.

Treatments	CR+PR,n=51	CR,n=32	PR,n=19	NR,n=21	*P*1	*P*2
MP Pulse Therapy (%)	7(13.7)	6(18.8)	1(5.3)	1(4.8)	0.239	0.492
Glucocorticoid(%)	43(84.3)	27(84.4)	16(84.2)	9(42.9)	0.002*	<0.001*
IVIG(%)	7(13.7)	4(12.5)	3(15.8)	1(4.8)	0.570	0.492
Cyclophosphamide (%)	6(12)	4(12.9)	2(10.5)	3(14.3)	1.000	1.000
Cyclosporine (%)	7(13.7)	3(9.4)	4(21.1)	0	0.079	0.177
Tacrolimus (%)	4(7.8)	1(3.1)	3(15.8)	0	0.084	0.315
Hydroxychloroquine (%)	31(60.8)	19(59.4)	12(63.2)	7(33.3)	0.102	0.034*
Platelet Transfusion (%)	12(23.5)	4(12.5)	8(42.8)	3(14.3)	0.042*	0.576

*P < 0.05. *P*1 represents the analysis of differences among the CR, PR, and NR groups, while *P*2 represents the analysis of differences between the CR+PR and NR groups. MP: methylprednisolone, IVIG: intravenous immunoglobulin.

### 3.3 Logistic regression analysis

Variables with P-values <0.05 in the univariate analysis, including age, complement 3 levels, PLR, NLR, and SII, were included in the logistic regression model. Both the Omnibus test and the goodness-of-fit test indicated that the model was statistically significant and had satisfactory goodness-of-fit, with a Hosmer-Lemeshow (H-L) test P-value of 0.409.

Logistic regression analysis revealed the following significant associations: NLR was positively correlated with treatment efficacy (odds ratio [OR]=1.982, 95% confidence interval CI: 1.18–3.33, *P*=0.010); SII was negatively correlated with treatment efficacy (OR=0.991, 95% CI: 0.984–0.998, *P*=0.011); Complement 3 levels were negatively correlated with treatment efficacy (OR=0.045, 95% CI: 0.002–0.919, *P*=0.044). Detailed results are summarized in **Table 4**.

**Table 4:** Logistic regression analysis of SLE-ITP treatment outcomes.

Variables	B Coefficient	*P*-Value	Odds Ratio	95% Confidence Interval
NLR	0.684	0.010*	1.982	1.18-3.33
SII	-0.009	0.011*	0.991	0.984-0.998
C3,g/l	-3.098	0.044*	0.045	0.002-0.919

*P<0.05. NLR, Neutrophil-to-lymphocyte ratio; SII, Systemic immune-inflammation index (platelet count × neutrophil count/lymphocyte count); C3, Complement 3.

### 3.4 ROC curve analysis and prediction of treatment response outcomes

**Figure 1:**
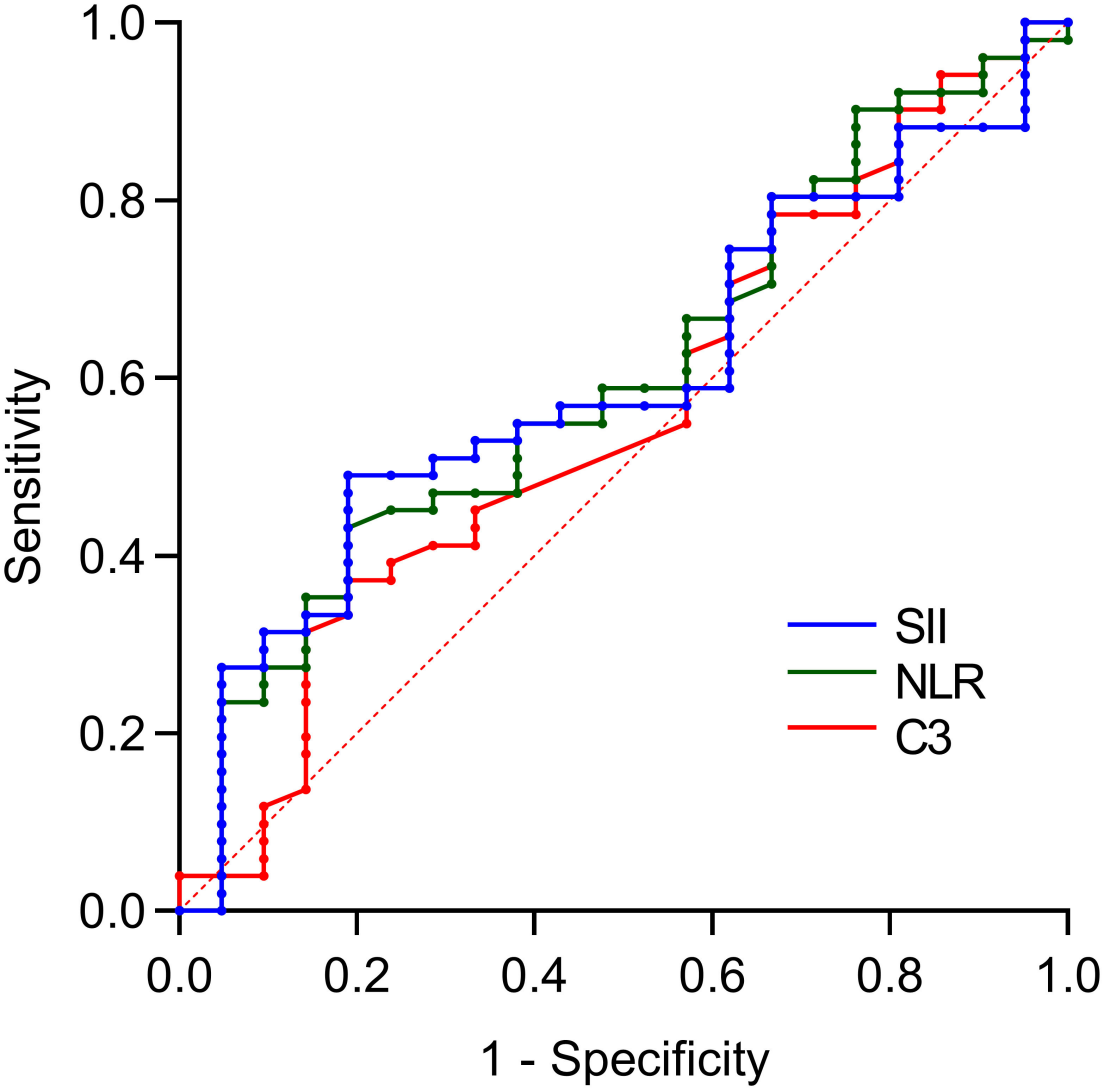
Predictive value of single variables for treatment efficacy. The predictive performance of individual biomarkers was as follows: NLR (AUC = 0.602, 95% CI: 0.462–0.743), SII (AUC = 0.599, 95% CI: 0.462–0.737), and complement C3 (AUC = 0.561, 95% CI: 0.414–0.707).

Based on the results of the multivariate logistic regression analysis, statistically significant variables (NLR, SII, and complement 3) were included in the receiver operating characteristic (ROC) curve analysis. Individual analysis demonstrated that the area under the curve (AUC) for NLR, SII, and complement 3 in predicting SLE-ITP treatment efficacy was not statistically significant, as shown in **Figure 1** and **Table 5**.

**Table 5:** Application of experimental inflammatory markers in predicting SLE-ITP treatment efficacy.

Test variables	AUC	*P*-value	95%CI
NLR	0.602	0.173	0.462-0.743
SII	0.599	0.187	0.462-0.737
C3,g/l	0.561	0.421	0.414-0.707
NLR+SII	0.671	0.023*	0.539-0.803
SII+C3	0.565	0.389	0.419-0.711
NLR+C3	0.621	0.109	0.476-0.765
SII+NLR+C3	0.743	0.001*	0.620-0.866

*P<0.05 indicates statistical significance; NLR, Neutrophil-to-lymphocyte ratio; SII, Systemic immune-inflammation index (platelet count × neutrophil count/lymphocyte count); C3, Complement 3.

**Figure 2:**
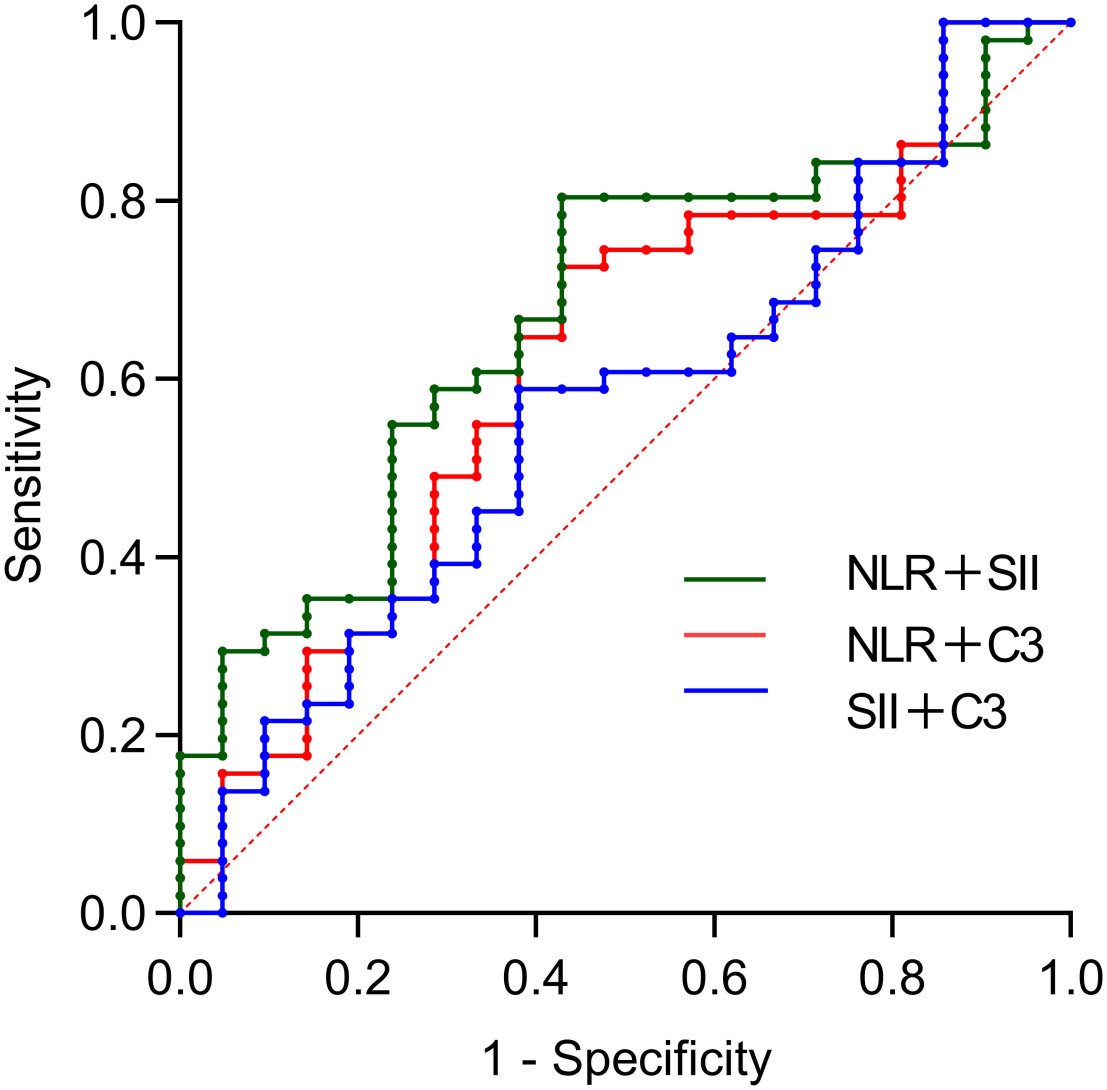
Predictive value of bivariate combinations for treatment efficacy. The AUCs were: 0.671 (95%CI: 0.539-0.803) for NLR+SII, 0.565 (95%CI: 0.419-0.711) for C3+SII, and 0.621 (95%CI: 0.476-0.765) for C3+NLR.

Further combined analysis revealed that the combination of NLR and SII significantly predicted SLE-ITP treatment efficacy, with an AUC of 0.671 (95% CI: 0.539–0.803, *P*=0.023). However, the combination of C3 and SII (AUC=0.565, 95% CI: 0.419–0.711, *P*=0.389) and the combination of C3 and NLR (AUC=0.621, 95% CI: 0.476–0.765, *P*=0.109) did not achieve statistical significance, as illustrated in **Figure 2** and **Table 5**.

**Figure 3:**
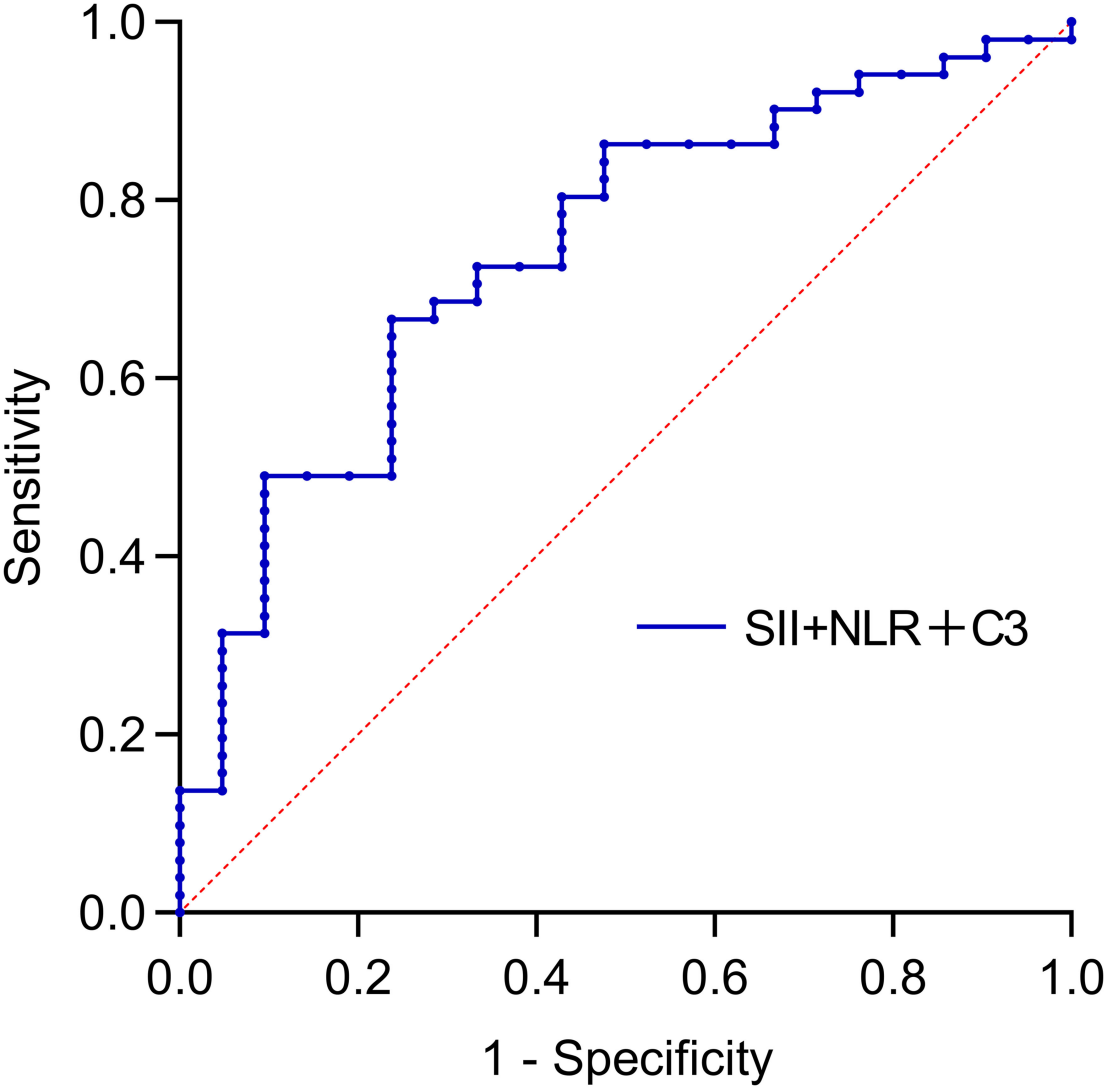
Predictive value of trivariate combinations for treatment efficacy. The AUC was: 0.743 (95%CI: 0.620-0.866) for SII+SII+C3.

This study not only utilized bivariate combined indicators for predicting SLE-ITP treatment response but also further explored the predictive efficacy of the combination of C3, NLR, and SII. The results demonstrated that the combined C3+NLR+SII index had significant predictive efficacy, with an AUC of 0.743 (95% CI: 0.620–0.866, *P*=0.001), an optimal cutoff value of 0.704, a specificity of 0.762, and a sensitivity of 0.667. This indicates that the C3+NLR+SII combined index provides accurate prediction and valuable clinical utility in SLE-ITP treatment response, as shown in **Figure 3** and **Table 5**.

### 3.5 Correlation analysis between SII/PLR and baseline platelet count in SLE-ITP patients

**Figure 4:**
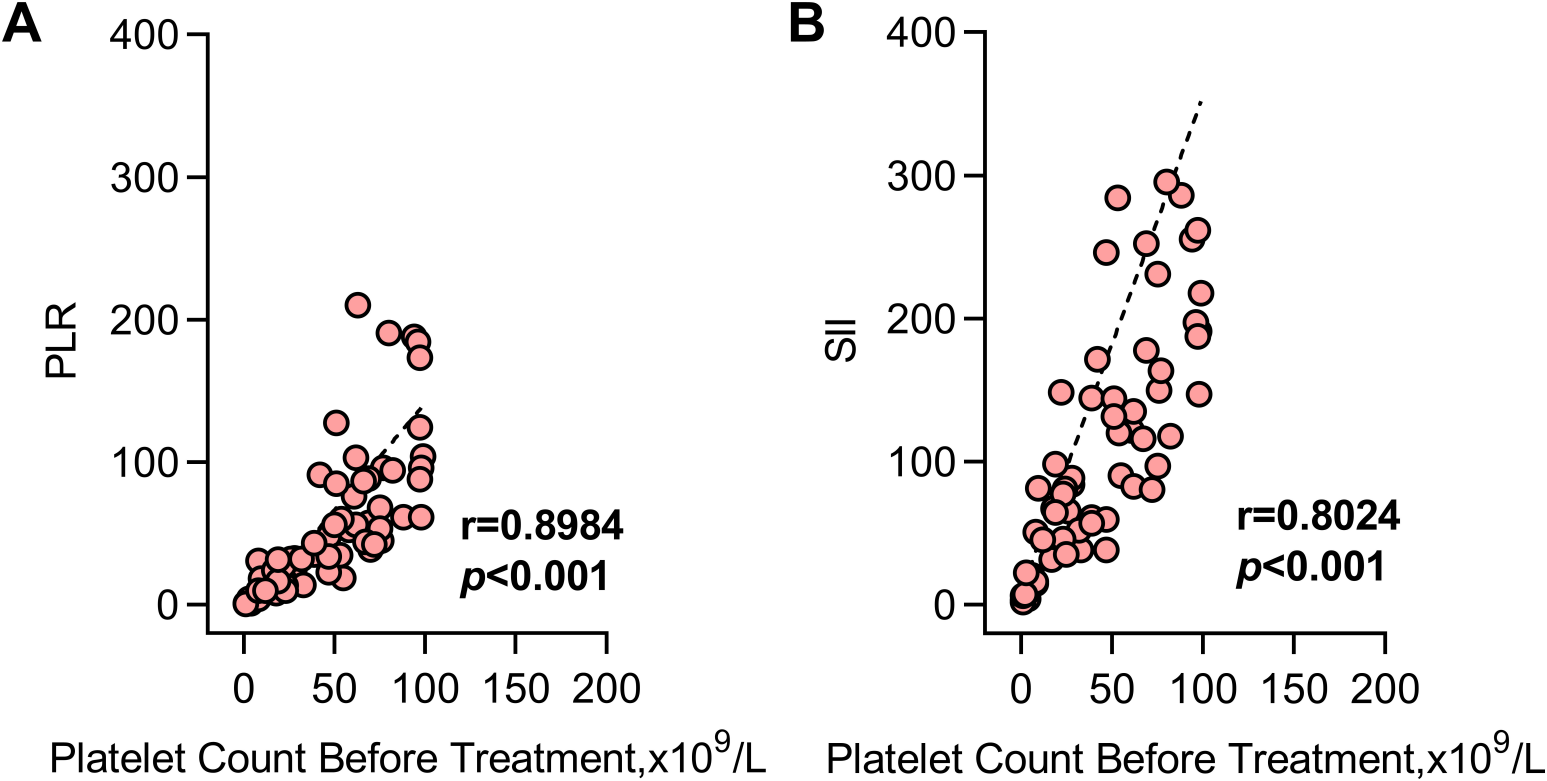
Correlation between SII/PLR and ITP. PLR showed a significant positive correlation with baseline platelet counts in SLE-ITP patients [r=0.8984, P<0.001; **(A)**]; Similarly, the SII level was positively correlated with baseline platelet counts [r=0.8024, P<0.001; **(B)**].

Spearman correlation analysis revealed significant positive correlations between both the PLR (r=0.8984) and SII (r=0.8024) with baseline platelet counts in SLE-ITP patients (both *P*<0.001), as illustrated in **Figure 4** respectively.

## 4 Discussion

The pathogenesis of SLE-ITP remains incompletely understood, involving complex immunoregulatory abnormalities, complement system activation, and increased platelet destruction. Clinically, there is an urgent need for suitable biomarkers to predict individual responses to treatment.

In the pathophysiological progression of SLE-ITP, platelets, lymphocytes, and neutrophils constitute critical immune effector cell populations. Their interactions play essential roles in modulating inflammatory responses and immune regulation (19). During the immune response in SLE-ITP, platelet-derived antigens are primarily presented to helper T cells by antigen-presenting cells (e.g., macrophages and dendritic cells) in the spleen, leading to B lymphocyte activation, proliferation, and differentiation into plasma cells. This process ultimately results in the production of platelet-specific autoantibodies and subsequent platelet destruction (20). The activation of the CD40L-CD40 signaling pathway plays a pivotal role in regulating T cell-mediated B cell activation, proliferation, and differentiation in this immunological process. Notably, platelets, as crucial immune effector cells, highly express CD40L on their surface, thereby contributing indispensably to these cellular interactions (21). Additionally, platelets release mitochondria and mitochondrial DNA, activating neutrophils and inducing neutrophil extracellular trap (NET) formation. This exposes autoantigens and further amplifies localized and systemic inflammatory cascades and aberrant immune responses (2). NLR, SII, and PLR reflect systemic inflammation and immune status more comprehensively and accurately by integrating the interactions among neutrophils, lymphocytes, and platelets.

SII, a novel systemic inflammation biomarker, has demonstrated significant clinical value not only in the prognostic assessment of various malignancies but also in the diagnosis and prognosis of systemic autoimmune diseases. Multiple population-based studies have confirmed that peripheral blood inflammation-derived indices, such as NLR, SII, and PLR, exhibit promising clinical utility for assessing disease activity and monitoring inflammation levels in SLE patients. These markers are also significantly associated with renal activity scores in lupus nephritis (22, 23). However, whether NLR and SII can predict treatment responses in SLE-ITP patients remains unknown. Building on this foundation, our study used multivariate logistic regression analysis to rigorously screen key clinical parameters influencing SLE-ITP treatment responses. The results revealed that NLR was positively correlated with favorable treatment outcomes, while SII and complement C3 were negatively correlated, suggesting that the latter two may be risk factors for poor efficacy.

Innovatively, our study employed bivariate combined indices to predict treatment responses in SLE-ITP patients and constructed the first combined predictive model integrating complement C3, NLR, and SII, significantly enhancing the predictive capability of individual indices. ROC curve analysis showed that the predictive efficacy of individual variables (SII, NLR, and C3) was moderate, while the combination of SII and NLR achieved improved predictive performance (AUC=0.671). To further optimize predictive accuracy, we developed a combined predictive model incorporating SII, NLR, and C3. The results demonstrated that this three-indicator model exhibited superior discriminative ability compared to individual indices and bivariate combinations, with an AUC of 0.743, indicating better clinical value. This prediction model’s current primary application lies in providing clinical warnings for SLE-ITP patients (e.g., intensive dynamic platelet monitoring), while its use for guiding drug dose adjustments or therapeutic modifications remains secondary and must be cautiously individualized. Therefore, pretreatment assessment of SII, NLR, and complement C3 in SLE-ITP patients may help predict treatment outcomes and prognosis, offering valuable guidance for clinical management.

This study holds significant clinical implications. First, it identified multiple novel clinical predictors of treatment response in SLE-ITP. Second, compared to single biomarkers, the combined predictive model (SII+NLR+C3) demonstrated enhanced performance in evaluating SLE-ITP outcomes, alongside its cost-effectiveness, convenience, and high patient acceptability. However, several limitations should be acknowledged. As a single-center, real-world retrospective study, the relatively small sample size may pose constraints in generalizing the findings. Given the limited number of patients treated with belimumab, rituximab, or mycophenolate mofetil in our study, we intentionally refrained from independent drug effect analysis to prevent potential bias from extremely small sample sizes. This primarily reflects resource constraints (e.g., economic factors) in regional hospital settings. Future multicenter, large-scale prospective cohort studies are needed to validate the conclusions of this research.

## 5 Conclusion

NLR was positively correlated with treatment efficacy in SLE-ITP, while SII and C3 were negatively correlated with treatment response. These factors provide novel biological markers for predicting SLE-ITP treatment outcomes. The combined predictive model constructed in this study, incorporating SII, NLR, and C3, significantly improved the predictive efficacy for SLE-ITP compared to individual indicators. This model demonstrates higher clinical application value and offers new insights for clinical diagnosis and treatment.

## Data Availability

The raw data supporting the conclusions of this article will be made available by the authors, without undue reservation.
